# ”It’s changed my life. I’m not smoking anymore. I don’t want to smoke anymore”: Exploring the Acceptability of Mailout Smoking Cessation Support for and by Aboriginal and Torres Strait Islander People

**DOI:** 10.1093/ntr/ntae065

**Published:** 2024-04-10

**Authors:** Kade Booth, Kayden Roberts-Barker (Wiradjuri), Joley Foster (Worimi), Amanual Getnet Mersha, Raglan Maddox (Bagumani), Billie Bonevski, Catherine Chamberlain (Palawa), Kerindy Clarke (Worimi/Gamilaroi), Catherine Segan, Michelle Kennedy (Wiradjuri)

**Affiliations:** College of Health Medicine and Wellbeing, University of Newcastle, Callaghan, New South Wales, Australia; Equity in Health and Wellbeing Research Program, Hunter Medical Research Institute, University of Newcastle, New Lambton, New South Wales, Australia; College of Health Medicine and Wellbeing, University of Newcastle, Callaghan, New South Wales, Australia; College of Health Medicine and Wellbeing, University of Newcastle, Callaghan, New South Wales, Australia; College of Health Medicine and Wellbeing, University of Newcastle, Callaghan, New South Wales, Australia; Equity in Health and Wellbeing Research Program, Hunter Medical Research Institute, University of Newcastle, New Lambton, New South Wales, Australia; National Centre for Epidemiology and Public Health, Australian National University, Canberra, Australian Capital Territory, Australia; Flinders Health and Medical Research Institute, College of Medicine and Public Health, Flinders University, Bedford Park, South Australia, Australia; Onemda Aboriginal and Torres Strait Islander Health and Wellbeing, Melbourne School of Population and Global Health, University of Melbourne, Melbourne, Victoria, Australia; Judith Lumley Centre, School of Nursing and Midwifery, La Trobe University, Melbourne, Victoria, Australia; Ngangk Yira Research Centre for Aboriginal Health and Social Equity, Murdoch University, Perth, Western Australia, Australia; Medibank Private, Sydney, New South Wales, Australia; Cancer Council Victoria, Melbourne, Victoria, Australia; Centre for Health Policy, Melbourne School of Population and Global Health, University of Melbourne, Melbourne, Victoria, Australia; College of Health Medicine and Wellbeing, University of Newcastle, Callaghan, New South Wales, Australia; Equity in Health and Wellbeing Research Program, Hunter Medical Research Institute, University of Newcastle, New Lambton, New South Wales, Australia

## Abstract

**Introduction:**

Aboriginal and Torres Strait Islander people want to quit smoking. There is global evidence of combination nicotine replacement therapy (c-NRT) alongside behavioral support as a best practice approach to smoking cessation care. However, there is limited adherence and acceptability research regarding NRT and behavioral supports for Aboriginal and Torres Strait Islander people. Similarly, there is limited research on what is considered culturally appropriate and safe support for Aboriginal and Torres Strait Islander people to quit smoking.

**Aims and Methods:**

This Aboriginal-led qualitative study explored the acceptability of the *Koori Quit Pack*. Aboriginal and Torres Strait Islander participants shared their experiences of quitting with the mailout c-NRT program and behavioral cessation support through Yarning. Collaborative Yarning and reflexive thematic analysis was used to develop themes.

**Results:**

Aboriginal and Torres Strait Islander people are motivated to quit smoking and have accessed cessation supports from health professionals. However, the support(s) received are not always appropriate or culturally safe. The *Koori Quit Pack* was deemed acceptable and useful for smoking cessation. Having access to smoking cessation care and knowledge of c-NRT helped people quit smoking and support others to quit too.

**Conclusions:**

A combination of NRT products alongside culturally responsive behavioral support(s), delivered through a mailout package was a beneficial strategy to help Aboriginal and Torres Strait Islander people quit smoking. National implementation of such a package could assist to accelerate reductions in tobacco use, helping meet national smoking reduction targets and improve health outcomes.

**Implications:**

Cessation supports offered to Aboriginal and Torres Strait Islander people are not always culturally safe or effectively delivered. The *Koori Quit Pack* provided Aboriginal and Torres Strait Islander people with culturally safe smoking cessation support delivered for and by Aboriginal and Torres Strait Islander people, demonstrating mailout smoking cessation supports as acceptable and highly valued. Mailout support eliminates accessibility barriers to cessation care while providing tools and knowledge to quit can lead to smoke-free behaviors among individuals and communities. Country-wide availability of this program can accelerate reductions in tobacco use, helping meet national targets and improve health outcomes consistent with the National Tobacco Strategy and the WHO Framework Convention on Tobacco Control.

## Introduction

Commercial tobacco was inflicted on Aboriginal and Torres Strait Islander people by European colonizers from 1788.^1,2^ Moreover, addiction related to commercial forms of tobacco use was deliberately manipulated to exploit Aboriginal and Torres Strait Islander people for economic and social gain, such as land and labor in lieu of monetary payment.^2,3^ Since this time, continuing racism and trauma stemming from colonization have contributed to ongoing tobacco use.^3^ Smoking is the leading preventable cause of death for Aboriginal and Torres Strait Islander people, causing over a third of all deaths,^4^ fueled by the Tobacco Industry.

Aboriginal and Torres Strait Islander people want to quit smoking,^5,6^ and there has been a continuing decrease in smoking rates among Aboriginal and Torres Strait Islander people in Australia.^7^ Despite being more likely to make a quit attempt than non-Indigenous Australians, Aboriginal and Torres Strait Islander people are less likely to sustain quit attempts.^8^ This clear eagerness to quit suggests that the high relapse could be attributed to a lack of appropriate supports, and therefore, demonstrates the need for increased cessation support strategies and programs that meet the needs of Aboriginal and Torres Strait Islander people.

The recently published Australian “National Tobacco Strategy 2023–2030”^5^ identifies reduced smoking rates for Aboriginal and Torres Strait Islander people as a key priority area. The strategy aims to “Ensure First Nations people have appropriate access to culturally safe cessation supports and subsidized nicotine replacement therapy, identifying, mitigating, and/ or addressing barriers to access and uptake of services supporting tobacco and nicotine cessation.”^5^ However, there is limited evidence to inform which cessation supports are “culturally safe” or to guide the uptake, access, and preference of nicotine replacement therapy (NRT) products as told by Aboriginal and Torres Strait Islander people.

The Royal Australian College of General Practitioners (RACGP) strongly recommends the use of combination NRT (c-NRT; ie, patches plus an oral form), alongside behavioral support for smoking cessation.^9^ However, as tobacco science has consistently privileged White Euro-Western experiences,^10^ there is limited research on the adherence and acceptability of NRT in conjunction with behavioral support for Aboriginal and Torres Strait Islander people. Previous research has reported low uptake of smoking cessation supports^11^; however, this has not been updated over time or complemented by qualitative evidence.

The lack of specialized research is expected to impact the ability to accelerate reductions in commercial tobacco use and meet the national reduction targets.

In Australia, the provision for General Practitioners (GPs) and GP-led telehealth counseling services to administer cessation care is funded under the Medicare Benefits Schedule (MBS), a list of services subsidized by the Federal Government.^12^ However, MBS subsidies are not available for Aboriginal Health Workers/Practitioners to support the implementation of a smoking cessation workforce into routine care,^13,14^ despite being well placed to provide it.

Quantitative studies show there are barriers for Aboriginal and Torres Strait Islander people to quit smoking despite wanting to, such as limited health provider support,^15^ and access, including cost of NRT.^11^ Qualitative research enhances understandings of how to mitigate and manage the challenges identified and is necessary to fill the current gaps in the research and translating findings into meaningful practice with positive results. Most qualitative studies on smoking cessation for Aboriginal and Torres Strait Islander people focus on barriers to quitting. There is minimal qualitative research that informs or evaluates successful cessation programs for Aboriginal and Torres Strait Islander people.

This article reports findings from qualitative interviews conducted to explore the acceptability of the *Koori Quit Pack*, a mailout smoking cessation support program, which included the provision of 10 weeks of free c-NRT to Aboriginal and Torres Strait Islander people. The program was developed in direct response to Aboriginal and Torres Strait Islander community needs, led by an Aboriginal research team, developed, implemented, analyzed, and governed in partnership with Aboriginal Community Controlled organizations in NSW and Victoria. Further details on the program can be found in the study protocol^16^ and additional outcomes papers.^17,18^

### Our Team: Who’s Doing the Research?

Relationality is foundational to Indigenous research practice and Yarning methods,^19^ as well as the responsibilities gifted through our respective knowledge and kinship systems. We recognize that our own lived experience and standpoint are vital to the research framing, development, conduct, and interpretation of findings. Our team embodies Aboriginal and Torres Strait Islander lived experience (MK, CC, KC, JF, KR-B), Indigenous lived experience (RM), expertise in Indigenous tobacco research (MK, CC, RM, AM, CS, BB), Aboriginal health services (MK), qualitative research (MK, KB), tobacco behavioral counseling (JF, BB, CS), smoking cessation research (MK, RM, KR-B, JF, AM, CS, BB, KB), and smoking cessation support (KR-B and JF). This study was designed to privilege Indigenous knowledge and recognize the ongoing scientific rigor of Aboriginal and Torres Strait Islander people since time immemorial.

### Governance

Aboriginal and Torres Strait Islander people were involved in all aspects of the study, from design to dissemination. The study was conceptualized and led by MK (Wiradjuri woman) and designed in partnership and co-owned with the Aboriginal Health and Medical Research Council (AH&MRC), Victorian Aboriginal Community Controlled Organization (VACCHO), and NSW/ACT and Victorian Quitlines. Partnering services have overseen the data collection, analysis, interpretation, and reporting to ensure appropriate community involvement. Preliminary findings were shared with Aboriginal Community Controlled Health Services across NSW and Victoria to help ensure interpretation, framing, and contextualization of the research were as accurate and as appropriate as possible.

## Methods

### Study Design

This qualitative study was developed by the Aboriginal research staff (JF, KR-B) and lead researcher (MK) to capture deeper insight into Aboriginal and Torres Strait Islander peoples’ experiences of quitting smoking. The stories shared during routine survey data collection time points contrasted with the published literature on the topic. This study used the Indigenous Standpoint^20^ of the research team, namely JF (Worimi), KR-B (Wiradjuri), MK (Wiradjuri), Indigenist Research Methodology^21^ and Yarning Method^22^ to privilege Aboriginal and Torres Strait Islander voices and experiences. The data collection, analysis, and interpretation were carried out by two Aboriginal research staff (JF and KR-B) who also supported participants in the wider pilot study, providing participant follow-up, free c-NRT, and behavioral support over 10 weeks. We recognize relationality, the relationships and trust formed between participants and the Aboriginal research staff as critical to upholding Indigenous knowledges, methodologies, and scientific excellence. By listening to the quitting stories of Aboriginal and Torres Strait Islander people using the *Koori Quit Pack*, we were able to gauge the acceptability of a mailout smoking cessation products and information, coupled with behavioral support.

### Relationship Between the Participants and the Researchers

Relationality is recognized and embedded through all aspects of our research process, informing the way the project has been designed, conducted, analyzed, and disseminated. That is, relationality to each other, our lands, stories, and knowledges, and practice.^19^ In line with Indigenist research methodologies,^21^ the Aboriginal researchers involved in this project not only collected data but also shaped what data was collected and why as driven by participants.

Two Aboriginal researchers (JF and KR-B) were responsible for recruitment and data collection. Relationships between the researcher and participants developed over the course of the study during routine follow-ups. Participants expressed a strong sense of comfort and relatability with JF and KR-B. Such rapport was crucial in how data collection was conducted, as Yarning is grounded in cultural positioning,^22^ where deeper relationality leads to greater thickness of data.^20^

The research questions were informed by the Aboriginal researchers’ conversations with participants during follow-ups, where the stories told were not being captured in past research or our quantitative component. Through Collaborative Yarning^22^ amongst the research team, domains of enquiry were developed as driven by participant stories and were a vital part of the conceptualization and development of the qualitative study. The domains were (1) stories of quitting, (2) perceptions of NRT during current and previous attempts, (3) experience with the *Koori Quit Pack* program, and (4) family and community. As Distinguished Professor Linda Tuhiwai Smith writes:

When Indigenous people become the researchers and not merely the researched, the activity of research is transformed. Questions are framed differently, priorities are ranked differently, problems are defined differently, people participate on different terms.^23^

The sentiment expressed above is deeply embedded through this study, and informed the results as guided by Aboriginal and Torres Strait Islander people.

### Participants and Recruitment

This research was part of a larger study that involved a baseline survey and a 10-week follow-up survey. Aboriginal and/or Torres Strait Islander people who smoked daily, aged 16 years and above, resided in NSW, ACT, or Victoria, and had a plan to quit smoking in the 30 days following enrollment to the program were eligible. Participants were able to elect permission to be involved in further projects during their baseline survey. From this, participants who completed the 10-week survey were contacted via text using the study phone, or email and offered the opportunity to partake in qualitative interviews about their experience with the *Koori Quit Pack*. A total of 14 Yarns were conducted over the phone.

### Data Collection

Yarning method was used as a means of allowing participants to share their story in a culturally safe, open and relaxed manner, producing richer and meaningful data.^22^ Through Yarning, the “researcher and participant journey together visiting places and topics of interest relevant to the research study.”^22^ Conversation began with a Social Yarn to touch base with participants and reestablish trust and rapport. Research Topic Yarning then followed to explain the research and gain consent before commencing the formal data collection. Twelve audio recorded Yarns were conducted over the phone at a time convenient to the participant by both researchers (KR-B and JF), with two additional conducted independently by KR-B. Following the *reflexive thematic analysis (TA)*^24^ approach, data saturation was not necessary or useful for this study.^25^ Rather, in line with the presented aims, we share the experiences and stories of participants to provide insights into preferred smoking cessation support for Aboriginal and Torres Strait Islander people and the acceptability of support delivered through *Koori Quit Pack.*

### Analysis

Once transcribed, the data were imported into Nvivo software for analysis. Yarns were coded by one non-Indigenous (KB) and one Aboriginal (KR-B) researcher using reflexive TA.^24,26,27^ KB is experienced in qualitative research and worked collaboratively alongside KR-B, who is new to qualitative research analysis, to provide training and two-way learning. JF was actively involved in discussions during the entire process as part of the collaborative analysis approach, including interpretation of the data by all those involved in collecting the data. This approach assisted to ensure participant stories were appropriately and accurately reported.^28^

Collaborative Yarning is a component of Yarning method, which involves Yarning among people discussing concepts, ideas, and information about a research project.^22^ As Bessarab and Ng’andu conceptualize: the “sharing of research findings can lead to new discoveries and understandings.” We utilized Collaborative Yarning as the primary method through all stages of the research including analysis. We also incorporated reflexive TA,^24,27^ of which the non-Indigenous researcher (KB) is experienced, as a secondary method that complimented the Collaborative Yarning. In this way, we prioritized Indigenous methods with the support of Western methods. In doing so, the analysis process privileged Aboriginal researchers who conducted the interviews and their relationality to participants and the research as the experts in meaning making. This approach was particularly useful for capability building of Aboriginal and non-Aboriginal team members, guiding a culturally responsive coding process, as well as collectively form meaning, and development of themes.

The coders familiarized with the data set during collection (KR-B) and reading transcripts (KR-B and KB). Ongoing discussion between the team strengthened the immersion process. The initial four Yarns were independently dual-coded (KR-B and KB) before meeting to discuss. This process was not used to ensure agreeance, but as part of the collaborative process, where we discussed meaning, reflected, and shared ideas. Any discrepancies between coders were reflexively discussed, and placed into separate codes as two coders were used for two-way learning, collaboration, and exploration of ideas rather than as a measure of “coding quality”.^29^

Although not typically used in reflexive TA, the coders developed a codebook. We found this a useful tool to help reflexively navigate meaning and guide learning. This was not used to “define” or “categorize,” but rather as a dynamic tool for reflection and discussion between coders. In total, nine were dual-coded (KB and KR-B), and five independently coded by KR-B with numerous meetings throughout the coding process and ongoing guidance and yarns with JF.

The researchers (KB, KR-B, and JF) met once coding had been completed to review findings and generate initial themes. The researchers then collaboratively developed and refined themes alongside MK by reviewing the coding and “telling an interpretive story about it.”^30^ Themes were considered to capture a core idea and convey participant stories.

### Ethics

Community-based approval and agreements were developed with partnering organizations to uphold data sovereignty principles.^31^ The project upholds ethical principles of research with Aboriginal and Torres Strait Islander peoples consistent with the National Health and Medical Research Council’s Guidelines for ethical conduct in Aboriginal and Torres Strait Islander health research,^32^ and the “AH&MRC Ethical Guidelines: key principles.”^33^ Ethical approvals were obtained from the AH&MRC Ethics Committee of NSW [1894/21] and the University of Newcastle [H-2022-0174]. All participants provided informed consent. The study is registered with the Australian New Zealand Clinical Trials Registry (#12622000654752). The reporting of the project has been conducted in line with the CONSIDER statement^34^ and ethical publishing practices.^35^

## Results

Fourteen participants shared their experiences of quitting smoking using mailout cessation support (see Table 1). Most had reportedly quit at the time of the Yarn and others had reportedly cut down significantly with intention to quit. Participants were eager to share their experience, as they considered the support given to aid their own quit attempt, also helped others to quit. While involved in the broader program, no Torres Strait Islander people participated in this qualitative study.

**Table 1.** Participant Demographics

ID		Age	Smoke-free for past 6 months	7-day smoke-free at 6 month	Intent to make future quit attempt
P01	Wiradjuri woman	53	No	✓	✓
P02	Worimi woman	59	✓	✓	—
P03	Ngunawal woman	35	✓	✓	—
P04	Gulidjan woman	51	No	No	✓
P05	Wathaurong woman	34	✓	✓	—
P06	Bundjalung man	47	No	No	✓
P07	Latje Latje woman	42	✓	✓	—
P08	Dharug woman	53	No	No	✓
P09	Wiradjuri woman	33	✓	✓	—
P10	Wonnarua woman	53	✓	✓	—
P11	Dharug man	53	No	No	✓
P12	Dharug woman	57	✓	✓	—
P13	Dharug woman	51	No	No	✓
P14	Awakabal woman	38	✓	No	—

Participants spoke of their quitting journey in terms of past and present experiences. The following section explains this through the four themes and subthemes derived from the yarns, and ultimately, what worked and was considered acceptable for smoking cessation as told by Aboriginal people. Illustrative quotes and adjoining participant information can be found at Table 2.

**Table 2.** Themes and Illustrative Quotes

Theme/subtheme	Illustrative quote
Theme 1: Quitting for “My family, my health, my mob^*^”	I’m 33 years old and my mum passed at 55. She had diabetes and she smoke and drank and done wild stuff. She passed away at 55. Then my older sister, she passed away at 29, and my dad passed away when he was 30. So, I’m kind of the oldest in the family, which is sad, I’m only 33, and I don’t want to do that to my kids.—P09, Wiradjuri, Age 33 It wasn’t really about money for me. It was about conquering this and bettering my health.—P04, Gulidjan, Age 51 There’s health stuff. Like I’ve lost a lot of family pretty young, like under 60, and I think that some of it is definitely smoking related. And I’m, you know, getting close to 50 now, so I’m thinking I’ve got to get a handle on this and just stop. Plus my kids.—P06, Bundjalung, Age 47 And because I’ve got the magnet on the fridge, it’s like all the other reasons too, my family, my health, you know, my mob.—P10, Wonnarua, Age 53
Theme 2: “I’ve never had anyone really give a toss”: There is insufficient culturally safe smoking cessation support	In the past, I’ve never had anyone really give a toss if I give up or not. Usually, I’m going to a community group, they throw you a couple of packs of this and they say, try this. And that’s it, that’s the end of it.—P11, Dharug, Age 53 I got some free patches from [my local] Aboriginal medical service through appointment there, but she sort of just gave me a script for them to pick up and that was it. I tried one on and I didn’t like it. And that was it.—P09, Wiradjuri, Age 33
Subtheme 2.1: Past cessation support has been limited and not tailored to individual needs	The doc was just prescriptions and the Quitline were often busy, hard to get hold of. And you couldn’t text them. So, I found it a bit trickier to get hold of them.—P05, Wathaurong, Age 34 And she’s a good doctor but, yes, it’s always rushed. And you feel like you can’t talk about strategies, you can’t talk about having the barriers and all that kind of stuff. So, it’s not in-depth.—P14, Awabakal, Age 38 Because with the doctor, she just gave me a script and said, “On your way and good luck,” whereas you guys sort of reached out every couple of months or weeks and that was really good.—P06, Bundjalung, Age 47
Subtheme 2.2: There is inadequate access to combination NRT products and information	And knowing I could use the two together. I think if it was just the one, like if I was just using patches and not something else, I don’t think I would have been able to give up.—P09, Wiradjuri, Age 33 And I never used two types, nobody suggests that you use two types of NRT. That wouldn’t have dawned on me. So, it was Koori Quit Pack suggesting that and saying that they’d work with me.—P12, Dharug, Age 57 I’ve definitely had so much information with all the paperwork, all the booklets that you put in the packs was so good. I never got that anywhere, for anything.—P04, Gulidjan, age 51.
Subtheme 2.3: NRT products are not affordable or accessible	See, I only ever went cold turkey because I could never afford none of the replacement therapy stuff. So, it never really lasted.—P07, Latje Latje, Age 42 And I tried the lozenges, but they just made me want to throw up, they tasted dreadful. And, yes, that’s probably as far as I went because I, kind of, went, oh, God, that’s twice I’ve tried this stuff and it’s expensive.—P05, Wathaurong, Age 34 I think we spoke last time, too, about with the government or the PBS thing how the doctor only can prescribe two or three months’ worth and that was it for 12 months. And for people like myself, I don’t think that’s enough.—P06, Bundjalung, Age 47 I could not afford to pay all my NRT on my income because I’m only a low-income earner. I wouldn’t have the opportunity if it wasn’t for you guys.—P01, Wiradjuri, Age 53
Theme 3: Having access to smoking cessation care: “It’s changed my life”	It’s changed my life. I’m not smoking anymore. I don’t want to smoke anymore.—P09, Wiradjuri, Age 33 And then the doctor actually had to start putting me on the nebulizer. And now I’m back to actual normal just puffers instead of being on a nebulizer four times a day.—P07, Latje Latje, Age 42 Because I go with swimming with my daughter and my grandson every weekend. And my daughter goes, “It’s so much nicer that we don’t walk out of the pool and you’re instantly looking for your smokes, Mum.”—P05, Wathaurong, Age 34 It’s a huge change. Well, I’m nearly four months without a cigarette now.—P07, Latje Latje, age 42. So, yes, you haven’t just helped me, you’ve helped a couple of friends and kids that I’ve worked with.—P05, Wathaurong, age 34.
Subtheme 3.1: Mailout cessation care is acceptable and preferred	But it’s more so doing it in an environment you’re already comfortable with, without the added pressure of having to go somewhere to seek out the help.—P03, Ngunawal, Age 35 You don’t hassle if you haven’t given up smoking. Whereas if you go to the doctor, you get a lecture, oh, you haven’t given up smoking. You guys don’t add to the stress; you take it away.—P01, Wiradjuri, Age 53 And the fact that the NRTs were delivered to the house as well. Again, there’s an element of shame having to go to a chemist to by something which is really irrational. Because no one there is going to judge you, but I suppose in the past, I judged myself and then think other people are judging me, and they’re not, they’re probably not. It’s just me being dumb having these stupid thoughts. But I suppose when there’s that element of shame to smoking which is sort of where I had gotten to, having everything being able to be delivered at homes it’s not somewhat keeping the battle of it private to quit. But it’s more so doing it in an environment you’re already comfortable with, without the added pressure of having to go somewhere to seek out the help.—P03, Ngunawal, Age 35
Subtheme 3.2: Choice and combination NRT enhanced quitting experiences	I don’t know, I think now, there was a bit more options and the opportunity to try everything was good with you guys. We had the option of what we wanted to try, what worked in the past, what didn’t.—P01, Wiradjuri, Age 53 Whereas with the chewing gum and the inhaler, I found that was very easy because it was looking for something to do with your hands and you just put that inhaler in. You had that in your hand. You put it in your mouth and pretended you were smoking.—P02, Worimi, Age 59 So, it just really hasn’t been successful in the past. Whereas this time around I suppose I never really realised prior to all this that using two different types of NRT and finding a combination that works is actually going to be the most successful and it seemed to actually take less effort I feel.—P03, Ngunawal, Age 35 But this time was very different because, one, I think I had a lot of different options, like the chewy, the inhaler, the bloody patch and the lozenges.—P08, Dharug, Age 53 And knowing I could use the two together. I think if it was just the one, like if I was just using patches and not something else, I don’t think I would have been able to give up.—P09, Wiradjuri, Age 33 And that’s when I reached out to Koori Quit Pack. And I’d actually started because I think I had some patches from one of the times I’d tried before. But I hadn’t used the combination of the patches and the lozenges that worked for me.—P12, Dharug, Age 57 I think the difference is I’ve had support. And not only that, you have helped me with products and helped me how to use the products correctly.—P04, Gulidjan, Age 51
Theme 4: Mob Supporting Mob— For us, by us—”That’s what Koori People do”	That’s what Koori people do, they like to build relationships with people and speak to other people from our culture or our mob, and that’s very important. That’s why the program’s been successful, because we are talking to mob.—P02, Worimi, Age 59 Knowing it was other Blackfellas and it wasn’t just White people telling you what to do and what they think you should do. Having someone who understood and was there, no judging. I didn’t feel judged, I think that’s a big thing.—P14, Awabakal, Age 38 So, I think that needs to be mentioned somewhere, that, to help other Blackfellas out. Yes. Then if people think, oh, shit, [P14] can do it, then surely I can.—P14, Awabakal, Age 38 It’s not just the NRT. I just felt that when you rung me it helped too, because at times I was having down days. And I had that extra support with you ringing me as well. I think incorporating that, the support from the human part and the support from NRT is very vital for me.—P04, Gulidjan, age 51. If you can just stop one person, then the next generation can see that.—P10, Wonnarua, Age 53

^*^”’Mob’ is a term identifying a group of Aboriginal and Torres Strait Islander people associated with a particular place or Country. ‘Mob’ is an important term for Aboriginal and Torres Strait Islander people, as it is used to describe who they are and where they are from. ‘Mob’ is generally used between Aboriginal and Torres Strait Islander people.” *Sourced directly from:*https://healthinfonet.ecu.edu.au^36^

### Quitting for “My family, my health, my mob”

Participants were motivated to quit smoking for their health and family. They were aware of negative health outcomes associated with tobacco and were eager to improve their health and well-being. Alongside health improvements, participants noted family, mob, and community as a catalyst for making a quit attempt.

Participants frequently referred to family and community as intertwined with all aspects of their quitting journey. Notably, participants wanted to initiate generational change and ensure they were around longer for their children and grandchildren.

### “I’ve never had anyone really give a toss”: There Is Insufficient Culturally Safe Smoking Cessation Support

All but one participant had made a quit attempt prior to the *Koori Quit Pack*. The vast majority had accessed smoking cessation care in the past. However, participants reported a deficit in appropriate and available support. Barriers to quit attempts were often attributed to the lack of ongoing assistance from health professionals, limited information on available NRT products and how to use them, and the affordability of such products.

#### Past Cessation Support Has Been Limited and Not Tailored to Individual Needs

Most participants had seen their local GP or health service to quit smoking. Despite suggesting a good relationship with their usual health practitioner, participants noted not receiving ongoing cessation support. Often these were attributed to the “rushed nature” of GP and healthcare settings, where participants were given a script for products without follow-up, information on how to use it, and how to manage potential side effects.

Such services were not deemed as personal as the support offered by the Aboriginal research staff, or to provide the same level of personalized information. Minimal tailored support and information was deemed a key reason that participants struggled to stay quit in previous attempts.

#### There is Inadequate Access to Combination NRT Products and Information

Despite being offered NRT in the past when accessing cessation care, participants frequently said that they were given limited information on how to use products, manage cravings, and mitigate side effects. Most notably, participants were not aware that they were able to use multiple products through c-NRT or the range of products available to them.

#### NRT Products Are Not Affordable or Accessible

Cost was identified as a major barrier to accessing and adhering to NRT. The price of NRT was seen to inhibit quitting attempts, with some participants trying to quit cold turkey as a result.

Some stated that they simply were not able to afford NRT products, as they were on disability support pensions or low-income earners. For those who had used NRT, cost reduced their willingness to try again after past attempts led to adverse reactions.

Participants were wary to put out the large financial outlay to begin using NRT, and particularly c-NRT, with fears the attempt would be unsuccessful or the chance they would not find the purchased product to be suitable for their needs. This reluctance was particularly driven by past experiences of quitting with limited support and knowledge of products.

### Having Access to Smoking Cessation Care: “It’s changed my life”

Participants reported successful outcomes associated with the NRT mailout coupled with support from Aboriginal staff. Overall having access to appropriate support, products, and information delivered through a mailout package aided in overcoming the barriers identified in former quit attempts.

#### Mailout Cessation Care Is Acceptable and Preferred

Participants appreciated the NRT products being sent to their door and being able to register online through Facebook recruitment. Mostly, people enjoyed being able to access these products from the comfort of their own home. Participants reported feelings of reduced anxiety and “shame” associated with purchasing NRT products from a pharmacist or through a health professional as there is stigma around smoking.

Participants also suggested that having it arrive at your home reduced the stress or feelings of “letting down” their usual health practitioner due to potential relapse. Having the support from people that they did not necessarily know outside of the program helped remove these barriers for a “less threatening” process.

#### Choice and Combination NRT-Enhanced Quitting Experiences

The use of c-NRT was a fundamental difference between the current and past quit attempts. The program exposed participants to an array of products that they previously did not know were available to them. Once given the opportunity to try a tailored combination of products, participants reported having greater success in navigating triggers and ultimately a more successful quit attempt (ie, it made a hard job easier, not easy).

Once participants were given information on how to use the products, they were able to utilize them in meaningful ways. For example, participants who often smoked in their car found the sprays useful to manage urges and some reported using lozenges alongside patches to navigate “in-between” cravings.

Additionally, the mailout contained products that were usually financially inaccessible for participants. Participants emphasized the benefits of being able to try different products to see what worked for them without relying on considerable financial outlay. With the opportunity to try a variety of products to find ones suitable to their needs combined with information on how to use the products and ongoing support, participants reported a much greater chance at cessation success.

### Mob Supporting Mob— For Us, By Us—”That’s what Koori People do”

Participants frequently expressed the advantage of having culturally responsive support from Aboriginal researchers. The ongoing relationships developed between the participants and researchers were emphasized as a crucial component to quitting success and acceptability of the supports.

Participants reported improvements in their own health and well-being, which were often related to family and being able to spend more positive time with friends, children, and grandchildren.

Participants recognized the knowledges and supports associated with the mailout to benefit themselves, as well as an opportunity to share and support others to quit smoking.

In this way, mailout c-NRT alongside culturally responsive behavioral supports were perceived to build the skills and knowledge of Aboriginal people to change their own smoking behaviors, as well as empowering them to assist the next generation to quit and stay smoke-free.

## Discussion

In line with the newly released National Tobacco Strategy 2023–2030, this Aboriginal-led and governed study aimed to address the questions of “what is culturally safe cessation support?” and “what is the acceptability of a mailout smoking cessation package for Aboriginal and Torres Strait Islander people?” In measuring the acceptability of such a package, we both *identify* and *address* barriers to cessation services and detail successful cessation supports. This study found that Aboriginal people are motivated to quit smoking and have accessed cessation support. However, the support was not deemed efficient in supporting quit attempts. Participants found the *Koori Quit Pack* to be an acceptable and a preferred support method, which gave them the necessary tools to quit smoking and help others to be smoke-free.

Research indicated that Aboriginal and Torres Strait Islander people are less likely to have used NRT products than non-Indigenous Australians (37% vs 58.5%).^11^ However, our research has shown that Aboriginal people who smoke and want to quit *have* accessed NRT support through their usual health service. While barriers to NRT acceptability were not found in our study, participants did report previous experiences of inadequate information provided to them on how to appropriately use the products and limited knowledge on what products were available (ie, c-NRT, including oral and inhaler products). Such findings indicate that Aboriginal people want to use NRT to quit smoking, but it *must* be coupled with appropriate support and information.

Our findings align with past studies, which show a more receptive response to cessation supports when it is delivered in a positive,^37^ flexible,^38^ and culturally safe manner, without pressure^38^ and shame.^39^ This study demonstrated that an Aboriginal-led program including mailout c-NRT combined with culturally safe and responsive support not only supported Aboriginal people to achieve their quitting goal but empowered participants by providing the tools and knowledge to quit smoking, which ultimately can lead to smoke-free behaviors among families and communities.

Participants in our study found that a combination of NRT products alongside culturally responsive behavioral support was a preferred and beneficial strategy to successfully quit smoking. The RACGP guideline states that c-NRT accompanied by behavioral support is the recommended standard of nicotine dependence treatment.^9^ However, it is essential that a smoking cessation program is culturally safe and responsive,^3^ as well as meaningful and relevant to Aboriginal and Torres Strait Islander communities to be effective.

Participants in our study did not report receiving RACGP recommended care in previous quit attempts, although they had attempted to access it. Participants reported facing barriers related to health provider timeframes and a lack of support and information on how to correctly use or plan quit attempts. While global evidence for effective tobacco control in Indigenous communities is somewhat limited, we know that Indigenous leadership, partnership, engagement, and cultural tailoring are recommended^40^ and recognized as a human right.^41^ Programs to support Aboriginal and Torres Strait Islander people should be driven for and by Aboriginal and Torres Strait Islander people, and participate in the development, implementation, and evaluation of tobacco control and issues affecting them, as stated by the World Health Organization and the United Nations.^41,42^

The relationality between participants and Aboriginal project staff was highlighted as a key component in this study. We recommend that smoking cessation support services for Aboriginal and Torres Strait Islander people be delivered by Aboriginal and Torres Strait Islander people and based in an Aboriginal Community Controlled setting, which is known to be culturally safe and responsive.^43^ The utilization of text messages for follow-up appointments and to ensure participants had access to the research team for behavior counseling was considered highly feasible and appreciated by participants. Text messages should be considered in prevention and cessation programs to address barriers to accessing and adhering to Aboriginal and Torres Strait Islander smoking cessation supports.^44^

c-NRT products were associated with cessation success and particularly useful to manage personal triggers and navigate challenging scenarios. The success of c-NRT is evidenced internationally.^45,46^ Despite the clear benefits associated with c-NRT, not all products are government-subsidized on the PBS as combination.^47,48^ Gum and lozenge products are no longer available on the PBS, meaning there are currently no oral forms of NRT currently available on the PBS.^49^ The National Tobacco Strategy aims to review “restrictions on and the accessibility of current smoking cessation pharmacotherapies available on the PBS in the context of the latest evidence, best clinical practice, cost-effectiveness, and consumer affordability, and enhance the availability of these medications,”^5^ which shows a commitment to provide combination products on the PBS. We welcome this, as participants reported financial barriers to accessing c-NRT as impacting quit attempts. Access to oral forms of NRT for Aboriginal and Torres Strait Islander people is critical to uptake and adherence of c-NRT and increased likelihood of smoking cessation. This study emphasizes that a culturally responsive cessation package and support program delivered by Aboriginal and Torres Strait Islander people can help achieve these national targets and ultimately improve health outcomes.

Aboriginal and Torres Strait Islander people want to quit smoking^5,6^ and are motivated to quit not only to improve their own health but also to be role models for their children, families, and communities.^50,51^ Our research affirms and extends this sentiment. Participants in this study were motivated to quit for mob and community but were also motivated to help others quit when given appropriate supports. Once given the appropriate tools, they were driven to share this knowledge with community, help others quit, and be the change for future generations. Motivation to quit was experienced by the participants, but also reflected the authentic drive, connection, and relationality of the Aboriginal research staff. Relationality was foundational to this program, which was recognized by participants as a key component to their acceptance of the support offered. Relationality is also central to the Indigenous research paradigm^52^ that guides the research practice. This study demonstrates the strength of an Aboriginal designed, led, and delivered cessation support program in empowering individuals and communities to be smoke free.

COVID-19 changed the provision of health care services, including the delivery of health care services to Aboriginal and Torres Strait Islander communities. Some of these responsive and adaptive approaches have been evaluated and found to be effective.^53,54^ Acknowledging the national targets and current prevalence rate of 40.2%^7^ of Aboriginal and Torres Strait Islander people smoking, we recommend drawing on novel and community-led strategies utilized during COVID-19 to offer cessation support. It is critical that these are urgently funded and implemented to uphold Indigenous rights to health as determined by international law.

That’s what Koori people do, they like to build relationships with people and speak to other people from our culture or our mob, and that’s very important. That’s why the program’s been successful, because we are talking to mob.—P02, Worimi, Age 59

## Conclusions

Access to health is a human right. We know the harms that smoking has caused and continues to cause for Aboriginal and Torres Strait Islander people, and all Australians. Delivering tobacco prevention programs and smoking cessation supports is essential in upholding and protecting Aboriginal and Torres Strait Islander people’s right to health as aligned with international human rights laws.^41,42^ Aboriginal and Torres Strait Islander people are motivated to quit smoking and are accessing the available support. However, this study reports that available supports are not always appropriate, culturally safe and responsive, or effectively delivered. The current available supports have engrained barriers that increase challenges experienced by Aboriginal and Torres Strait Islander people in their quit attempts. Here, we present an Aboriginal-led, community governed and delivered, culturally responsive solution as told by Aboriginal people. Through the offer of a mailout smoking cessation package, Aboriginal participants in this study found a combination of NRT products alongside culturally responsive behavioral support as an accepted and beneficial strategy to successfully quit smoking. National availability of a mailout program can assist in meeting the national smoking reduction targets, international human rights laws, and ultimately lead to greater health outcomes for Aboriginal and Torres Strait Islander people but requires urgent funding and implementation.

## Data Availability

The data underlying this article cannot be shared publicly due to ethical requirements and the privacy of individuals that participated in the study.
